# LGR4 (GPR48): The Emerging Inter-Bridge in Osteoimmunology

**DOI:** 10.3390/biomedicines13030607

**Published:** 2025-03-02

**Authors:** Wonbong Lim

**Affiliations:** 1 Department of Orthopaedic Surgery, Chosun University, Gwangju 61453, Republic of Korea; wonbong@chosun.ac.kr; Tel.: +82-62-230-6193; Fax: +82-62-226-3379; 2 Laboratory of Orthopaedic Research, Chosun University, Gwangju 61453, Republic of Korea; 3 Regional Leading Research Center, Chonnam National University, Yeosu 59626, Republic of Korea; 4 Department of Premedical Program, School of Medicine, Chosun University, Gwangju 61452, Republic of Korea

**Keywords:** leucine-rich repeat-containing G-protein-coupled receptor 4, receptor activator of nuclear factor kappa-B ligand, osteoclasts, osteoporosis, osteoprotegerin

## Abstract

Leucine-rich repeat-containing G-protein-coupled receptor 4 (LGR4), a member of the G-protein-coupled receptor (GPCR) family, has been implicated in various regulatory functions across multiple differentiation stages and numerous target sites in bone diseases. Therefore, LGR4 is a potential regulator of nuclear factor-κB ligand (RANKL) during osteoclast differentiation. However, a comprehensive investigation of its functions and applications in bone immunology is lacking. This review discusses the molecular characteristics, signaling pathways, and role of LGR4 in osteoimmunology, with a particular focus on its interactions with RANKL during osteoclast differentiation, while identifying gaps that warrant further research.

## 1. Introduction

The skeletal and immune systems are fundamentally interconnected because of their shared stem cell hierarchies [1]. Bone marrow is the primary hematopoietic organ, providing a specialized environment conducive to the growth and differentiation of hematopoietic stem cells (HSCs), which are progenitors of immune cells [2,3,4]. This relationship establishes the structural foundation for regulating the immune system in accordance with the skeletal environment [4]. Cells from immune and skeletal tissues occupy the same spatial niche and engage in extensive interactions within the bone marrow cavity [5]. The term “osteoimmunology”, introduced by Aaron et al., underscores the interrelationship between the skeletal and immune systems, thereby linking bone metabolism to immune function [6]. Bone and the immune system are intricately associated with various regulatory molecules, including cytokines, chemokines, receptors, and transcription factors [7]. These molecules facilitate cooperative interactions between bone and immune cells, enabling the execution of essential functions, such as structural support, the regulation of mineral metabolism, and hematopoiesis [1]. The close relationship between the skeletal and immune systems has been highlighted in studies demonstrating that osteoclast-activating factors are secreted by immune cells in osteoimmune diseases, including rheumatoid arthritis (RA), periodontitis, and infections, leading to abnormal bone destruction [8]. Receptor activator of nuclear factor kappa-Β ligand (RANKL) is a pivotal osteoclast-activating factor that is closely associated with osteoimmunological systems [9]. Furthermore, RANKL is recognized for its multifaceted roles within both the immune and skeletal systems, including roles in the development of lymph nodes and the thymus and bone remodeling [10,11]. Leucine-rich repeat-containing G-protein-coupled receptor 4 (LGR4) is an additional receptor for RANKL during bone remodeling [12,13]. LGR4 competes with the canonical receptor RANK for RANKL binding during osteoclast differentiation and activation, thereby suppressing the RANKL-RANK-TRAF6 signaling cascade [14]. This inhibition results in the downregulation of NFATC1 activity, ultimately blocking RANKL-induced bone resorption [12]. Moreover, LGR4 activates the cAMP-PKA-CREB signaling pathway, which regulates the expression of Atf4 and osteocalcin in osteoclasts and bone sialoprotein and collagen type I in osteoblasts [15,16]. LGR4 deficiency causes delays in osteoblast differentiation while simultaneously increasing osteoclast activity, thereby influencing the dynamics of bone remodeling [15].

Despite its involvement in the bone remodeling cycle, recent studies have predominantly focused on the role of LGR4 in HSC differentiation [17]. Given the competitive nature of the HSC microenvironmental niche, the physiological significance of bones as lymphoid organs has garnered attention [18]. Research indicates that the activation of LGR4 by structurally modified receptors results in an increase in intracellular cyclic adenosine monophosphate (cAMP) levels and the subsequent activation of protein kinase A (PKA), which phosphorylates and activates the transcription factor cAMP response element-binding protein (CREB) [13]. While CREB is primarily recognized as a transcriptional activator, it can also function as a repressor when phosphorylated at Ser142 or when heterodimerized with co-repressor forms of the cAMP response element modulator and inducible cAMP early repressor [19,20]. CREB has been shown to negatively regulate the transcription of *CYR61* and nectin [21]. CREB may modulate CD14 expression through a feedback mechanism to maintain appropriate levels of responses to pathogen-associated molecular patterns [22]. Membrane-bound and soluble CD14 facilitate the recognition of invading pathogens by toll-like receptors (TLRs) and activate downstream signaling pathways, including those involving NF-κB and CREB [23]. An increase in cAMP/CREB activity subsequently reduces the expression level of CD14, thereby limiting the immune response to a suitable magnitude [24]. LGR4 functions as a co-regulator within the cAMP-PKA-CREB signaling pathway to sustain the equilibrium of immune responses [22]. The evidence presented herein suggests that LGR4 has potential therapeutic applications in the treatment of TLR-related septic shock and autoimmune diseases [25]. Identification of LGR4 as a regulator of innate immunity offers new insights into the physiological roles of this G-protein-coupled receptor in immune-related disorders [26].

This review presents a comprehensive overview of the activation and functional roles of LGR4 by synthesizing information regarding its known ligands and associated downstream signaling pathways in the context of osteoimmunology. Furthermore, evidence pertaining to LGR4 abnormalities across various bone and immune diseases, as well as in animal models and pathophysiological processes, is reviewed. It is imperative to identify the endocrine ligands of LGR4 and their practical implications. Consequently, a relationship between LGR4 and the interconnectedness of the skeletal and immune systems has been suggested, beginning with pioneering studies in osteoimmunology.

## 2. LGR4 Identification

**Figure 1:**
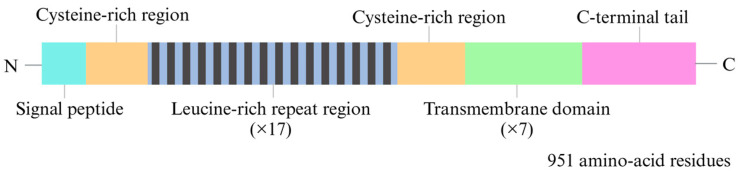
Illustration of the structure of the LGR4 protein. LGR4 is a transmembrane receptor. Its N-terminal domain comprises 17 leucine-rich repeat regions that are flanked by cysteine-rich regions. The protein has seven transmembrane domains and a C-terminal intracellular region.

LGR4 is a member of the G-protein-coupled receptor (GPCR) superfamily, which encompasses a distinct group of highly conserved proteins [26]. This receptor is a transmembrane protein that features an ectodomain containing multiple leucine-rich repeats (LRRs) that play critical roles in ligand binding [27]. The ectodomain is connected to seven transmembrane domains via a cysteine-rich hinge region that facilitates the activation of heterotrimeric G proteins [28]. LGR4 was characterized in 1998 because of its homology to well-established members of the LH/FSH/TSH receptor family [29]. The gene encoding LGR4 is highly conserved, spans 106,827 base pairs, and is located on human chromosome 11 (11p14.1). The gene comprises 18 exons that undergo alternative splicing, resulting in the production of two isoforms. One of these isoforms encodes 951 amino acids. Similar to other LGRs, LGR4 possesses a large N-terminal extracellular domain that facilitates binding to specific ligands [26]. The extracellular domain is characterized by 17 LRRs flanked by N- and C-terminal cysteine-rich regions [30]. Additionally, LGR4 contains seven typical transmembrane helical domains, three extracellular loops, three intracellular loops, and a C-terminal intracellular domain, which are common to all GPCRs (Figure 1) [28]

Phylogenetically, LGR proteins are categorized into three principal types [31]. Type 1 includes three hormone receptors: LGR1 (follicle-stimulating hormone receptor), LGR2 (luteinizing hormone receptor), and LGR3 (thyrotropin receptor). These receptors share a similar structural configuration characterized by a long hinge region and an ectodomain containing nine LRRs [31]. The type 2 receptor family, which includes LGR4, LGR5, and LGR6, is distinguished by the presence of additional LRR regions (16–18 LRRs) within the ectodomain [28]. Type 2 receptors are recognized for their critical roles in embryonic development and their involvement in the progression of various cancers. In contrast, LGR7 (RXFP1) and LGR8 (RXFP2), which have been identified as receptors for relaxin and insulin-like peptide 3, respectively, are type 3 LGRs [32,33]. These receptors are characterized by a short hinge region, low-density lipoprotein motif, and 10 LRRs. Recently, LGR5 and LGR6 have garnered significant attention because of their roles in stem cell differentiation, and they have been identified as specific stem cell markers in various adult tissues [34]. Additionally, R-spondin (RSPO) proteins act as functional ligands for this receptor class, demonstrating the capacity to enhance Wnt signaling through three type 2 LGR proteins [35]. Furthermore, LGR4, LGR5, and LGR6 are physically associated with LRP5/6 and Frizzled receptors, underscoring their vital roles in the Wnt signaling pathway [36].

LGR4 functions as a receptor for R-spondins (RSPOs), enhancing the canonical Wnt signaling pathway and contributing to the development of various organs [37]. Upon binding to RSPO1, RSPO2, RSPO3, or RSPO4, LGR4 associates with phosphorylated LRP6 and Frizzled receptors and is subsequently activated by extracellular Wnt receptors [38]. This activation initiates the canonical Wnt signaling pathway, leading to increased expression levels of target genes. Unlike traditional GPCRs, LGR4 does not activate heterotrimeric G-proteins [39]. Its role as an initiator of the Wnt signaling cascade is crucial for the development of multiple organs, including the liver, intestine, kidney, bone, and reproductive system organs [40]. Furthermore, LGR4 is integral to the activation of the Wnt signaling pathway in peripheral tubular myocytes during spermatogenesis and is essential for the maintenance of intestinal stem cells and Paneth cell differentiation within the intestinal villi postnatally [36]. Additionally, LGR4 plays a significant role in renal development during embryogenesis and in preserving the ureteral bud in an undifferentiated state [41]. It also functions as a negative regulator of innate immunity by inhibiting TLR2-/TLR4-mediated pattern recognition and the production of inflammatory cytokines [22].

The downstream signaling pathways associated with G protein activation have been well characterized [42,43]. Typically, hormones interact with the ectodomains of these receptors, leading to conformational alterations in the rhodopsin-like serpentine domain [44]. This process subsequently activates heterotrimeric G proteins, resulting in elevated intracellular levels of cAMP and the initiation of multiple signaling cascades [45]. The ligands and downstream signaling pathways associated with type 2 receptors, particularly LGR4, exhibit greater complexity than those associated with type 1 receptors [46]. This complexity may be attributed to structural differences and the presence of co-receptors between the two receptor types [47]. The number of LRRs and the spatial and structural modifications of LGR are thought to influence the binding specificity of ligands to LGRs, whereas coreceptors and intracellular domains modulate the activation of various signaling pathways. This study serves as a notable example of the integration of fundamental research into human genetics. However, this review does not adequately summarize the key characteristics of the distinct syndromes exhibited by LGR4 mutation carriers.

## 3. Regulation of Bone Formation

The skeletal system is characterized by a highly dynamic regenerative process that is predominantly regulated through modeling and remodeling [48]. During the developmental phase, the skeleton is established through the selective removal and deposition of bone at specific sites, a process referred to as modeling [49,50]. As the skeleton matures, a periodic regenerative process, known as remodeling, occurs, in which new bone tissue replaces older bone tissue [51]. This remodeling process is primarily facilitated by two distinct cell types: osteoblasts, which are responsible for bone formation, and osteoclasts, which are involved in bone resorption [18]. Osteoblasts are derived from bone marrow stromal cells (BMSCs), whereas osteoclasts originate from HSCs [52,53]. The equilibrium and regulatory mechanisms of these two cell types are crucial for maintaining bone homeostasis [54]. Dysregulation, characterized by the excessive activation of osteoclasts or the suppression of osteoblasts, can lead to bone disorders, such as osteoporosis [55]. Consequently, the identification of pathogenic genes that influence the development of osteoporosis has become a significant focus of research in this domain [56].

The CODE study was conducted to elucidate the relationship between variants of LGR4 and osteoporotic bone disease [57]. Analysis of metagenomic sequencing data from individuals residing in Iceland identified a rare nonsense mutation (p.R126X) in LGR4, which resulted in the premature termination of the LGR4 protein at amino acid position 126. The p.R126X mutation was first identified in the Icelandic genomic pool approximately 400 years ago and has not been observed outside Iceland [57]. Based on simulation studies, this mutation is predicted to disrupt the function of the receptor. The p.R126X mutation is closely associated with low bone mineral density (BMD) in the hip and spine. In another study, no mutations were detected at this site in a cohort of 11,748 Chinese individuals from the China Genomic Database [58]. Nevertheless, epigenomic and transcriptomic analyses have demonstrated a significant association between common single-nucleotide polymorphisms (SNPs) in *LGR4* and the risk of osteoporotic bone fractures [59]. Several studies conducted in Asian populations have indicated a correlation between specific SNPs in *LGR4* and individual bone quality [60,61,62]. Evaluation of the BMD phenotype in p.A750T knockin mice may provide insights into the functional relevance of these SNPs [63]. Further studies using mouse models are warranted to determine whether *LGR4* functions as a pathogenic gene in osteoporosis or high bone mass.

LGR4 is crucial for bone formation and remodeling [13]. It is constitutively expressed in BMSCs, HSCs, osteoblasts, and osteoclasts [64,65]. The downregulation of LGR4 inhibits the differentiation of BMSCs while promoting their proliferation; however, it simultaneously impedes their migration and induces apoptosis [66]. Both global and conditional knockout models of Lgr4 exhibit significant impairments in bone remodeling, which is attributed to LGR4-mediated regulation of the Atf4 signaling pathway through cAMP/PKA in osteoblasts [16]. Additionally, continuous mechanical stimulation has been shown to enhance the activation of Wnt/β-catenin signaling by LGR4, thereby promoting the osteogenic potential of RSPO1-stimulated BMSCs [67]. The RSPO2–LGR4 pathway enhances osteoblast differentiation by facilitating glycolysis [16,68,69]. Specific knockout of LGR4 in osteoblasts results in reduced bone strength [70]. Furthermore, LGR4 promotes the expression of pyruvate dehydrogenase kinase (PDK) 1, which induces aerobic glycolysis and lactate production, and regulates bone remodeling through the Lrp5–Gsk3β/β-catenin signaling pathway [16]. Notably, global LGR4 knockout upregulates the expression of *Pdk4*, a gene encoding a protein involved in fatty acid oxidation in muscle tissues [71]. Collectively, these findings suggest that LGR4 responds to various extracellular signals and nutrient stimuli, thereby activating multiple intracellular pathways to regulate bone remodeling.

Bone morphogenetic protein (BMP) is a member of a growth factor family within the TGF-beta superfamily, which plays a crucial role in osteogenesis [72]. In a study conducted by Mahasarakham et al., BMP2 treatment enhanced LGR4 expression both in vitro and in vivo [73]. Conversely, decreased LGR4 expression levels in osteoblasts partially inhibit BMP2-induced gene expression associated with osteocyte activation [74]. Notably, specific knockout of Lgr4 in uterine stromal cells results in a significant reduction in BMP2 expression levels [73]. This suggests that LGR4 signaling establishes a negative feedback interaction with BMP2. Furthermore, microRNAs (*miR-193a-3p* and *miR-137*) have been reported to bind to the 3′-untranslated region of *LGR4* mRNA, inhibiting the activation of transcription factors and thereby obstructing osteoblast differentiation [75].

**Figure 2:**
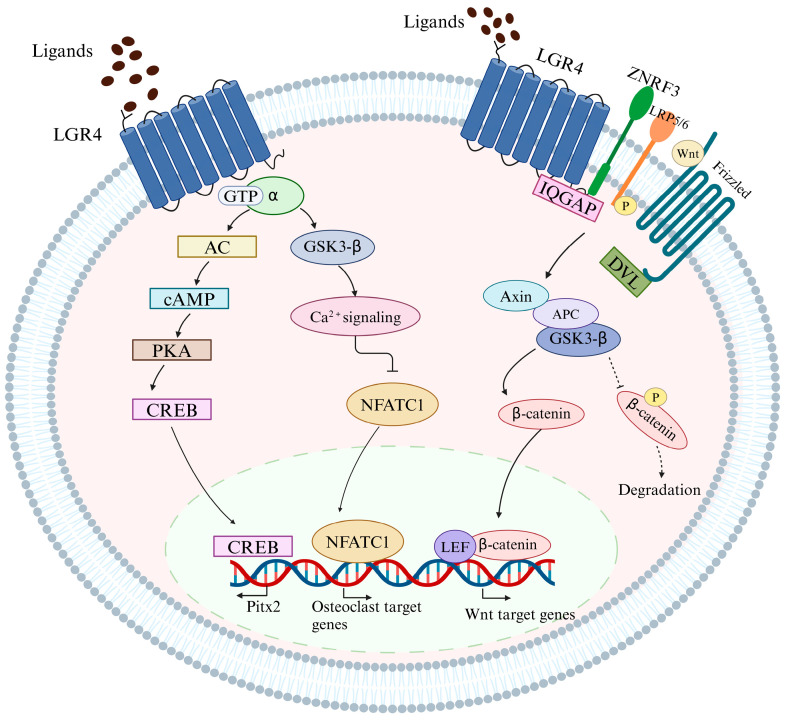
Intracellular signaling pathways of LGR4. As a classical GPCR, upon ligand binding to LGR4, it activates heterotrimeric G-proteins to transduce a cytoplasmic signal. After ligand binding, LGR4 activates several corresponding downstream signaling pathways, primarily the cAMP/PKA, Gαq/GSK3β, and Wnt/β-catenin signaling pathways. LGR4 mediates cAMP/PKA/CREB signaling by activating the heterotrimeric Gαs protein, which affects the transcription of target genes, such as *Pitx2*, *Atf4*, *CD14*, and *Esr1*. Meanwhile, LGR4 can interact with other ligands, such as RANKL, and activate Gαq, which causes intracellular Ca2+ release and suppresses the GSK3β-mediated activation of the nuclear translocation of NFATc1, eventually inhibiting osteoclastogenesis. In addition, RSPOs and Norrin have recently been identified as ligands of LGR4 that potentiate Wnt/β-catenin signaling. As ligands of LGR4, RSPOs potentiate Wnt/β-catenin signaling primarily via two methods. First, upon the binding of RSPOs to LGR4, ZNRF3/RNF43 is inhibited, and the Wnt/β-catenin signaling pathway is activated. The deubiquitinating enzyme USP42 can protect ZNRF3/RNF43 from ubiquitination and degradation and, thus, inhibit Wnt/β-catenin signaling. Second, in the presence of RSPOs, IQGAP1 is recruited by LGR4, and its affinity towards DVL is enhanced. Through IQGAP1–DVL interactions, a supercomplex with the Wnt signalosome is formed. IQGAP1 moves MEK1/2 to phosphorylate LRP5/6, which interacts with Axin and inhibits β-catenin phosphorylation.

Wnt/β-catenin signaling serves as a critical mediator in the process of bone formation. The binding of Wnt to Frizzled (FZD) activates the downstream β-catenin signaling cascade, with LRP5 and LRP6 functioning as co-receptors for FZD [76]. Mutations resulting in the loss of function of LRP5 are associated with osteoporosis, while gain-of-function mutations in LRP5 are linked to increased BMD [77]. RSPO1–4 proteins, secreted from corticosteroids, enhance Wnt/β-catenin signaling in mammals. RSPO1 and Wnt3a collaborate to induce the expression of osteoprotegerin (OPG) and facilitate osteoblast differentiation [78]. RSPO2 is vital for skeletal development, and it promotes osteoblast maturation and calcification. Mutations in RSPO2 can lead to severe limb defects [79]. RSPO3 is essential for the maintenance of cancellous bone and can be used to assess fracture risk in mice and humans [80]. Patients with RSPO4 mutations exhibit congenital anosmia or hyposmia [81]. However, the extent to which the effects of RSPO depend on LGR4 or other LGRs remains debatable. Nevertheless, RSPO2 promotes osteogenesis via Wnt signaling, a process that is diminished following the deletion of Lgr4, thereby confirming the essential role of LGR4 [68]. Additionally, RSPO3–LGR4 facilitates the differentiation of human adipose stem cells into osteoblasts [82]. Szenker-Ravi et al. reported that RSPO2 and RSPO3 can function as direct antagonistic ligands for RNF43 and ZNRF3 in the absence of signaling from LGR4, LGR5, or LGR6 [83]. These findings illustrate that the roles of LGR4, LGR5, and LGR6, along with those of their endogenous ligands, RSPO1, RSPO2, RSPO3, and RSPO4, do not entirely overlap but are redundant across different tissues, cell types, or developmental stages. There is a complex network of regulatory interactions among RSPO, LGR, LRP, ZNRF3/RNF43, and Wnt/β-catenin signaling in the context of bone metabolism (Figure 2). Although the RANKL and Wnt signaling pathways significantly contribute to this process, numerous gaps remain in our understanding of the pathogenic mechanisms underlying bone remodeling.

## 4. Regulation of Bone Resorption

LGR4 is a novel receptor for RANKL, which is capable of inducing the cAMP-PKA-CREB signaling pathways that regulate the expression of Atf4 and its target genes, including *Ocn*, *Bsp*, and collagen, in osteoblasts [15]. LGR4 is preferentially expressed in osteoblasts, and it plays a critical role in canonical Wnt signaling, which is essential for osteoblast formation and the maintenance of bone homeostasis. Furthermore, *miR-193a-3p* inhibits osteoblast differentiation by modulating the LGR4/ATF4 pathway [84]. Zhang et al. reported that the RSPO3-LGR4 signaling axis negatively regulates ERK/FGF signaling, thereby inhibiting the osteogenic potential of human adipose-derived stem cells [82]. Additionally, the application of a compressive force on the alveolar bone has been associated with the upregulation of RANK and downregulation of LGR4, which collectively promote osteogenic differentiation [85]. LGR4 is also crucial for the sequential development of molars via Wnt/β-catenin/LEF1 signaling pathways [86]. Silencing LGR4 has been shown to suppress both the proliferation and osteogenic differentiation of stem cells in the apical papilla by inhibiting the Wnt/β-catenin signaling cascade [87]. Arima et al. reported that the RSPO2-LGR4 signaling pathway promotes osteoblastic differentiation in immature human periodontal ligament cells [88]. Further investigations revealed a significant relationship between LGR4 and BMD, including its association with total fat mass [60]. Moreover, a rare nonsense mutation within LGR4 (c.376C > T) is strongly correlated with lower BMD and an increased incidence of osteoporotic fractures, as determined by whole-genome sequencing [89]. RNA sequencing analyses revealed that LGR4 is significantly differentially expressed between postmenopausal patients with impaired and normal BMD. Additionally, LGR4 deficiency can inhibit the differentiation of mesenchymal stem cells in bone marrow, leading to reduced bone mass and impaired fracture healing [90]. LGR4 induces the expression of PDK1 via canonical Wnt/β-catenin signaling [16]. Loss-of-function experiments have demonstrated that LGR4 deficiency leads to a reduction in osteogenic effects and aerobic glycolysis. Collectively, these studies highlight the significant mechanisms by which LGR4 influences bone differentiation and development, indicating its potential therapeutic applications in the treatment of osteolytic diseases.

LGR4 competes with the canonical receptor RANK for binding to RANKL during osteoclast differentiation [12]. RANKL is a critical regulator of osteoclast differentiation [91,92]. Historically, RANK was regarded as the sole receptor for RANKL. However, LGR4 has since been shown to directly interact with soluble RANKL and competitively inhibit its binding to RANK, thereby obstructing RANK-mediated osteoclast differentiation [12]. Observations in LGR4-knockout mice revealed abnormal osteoclast characteristics, including hyperactivity, increased proliferation, and reduced apoptotic features [12]. Notably, the administration of soluble LGR4-ECD, which binds RANKL, effectively mitigates bone loss in the femurs of ovariectomized mice and severe bone resorption in OPG-knockdown mice. Furthermore, LGR4, which is targeted by *miR-34c*, activates the NF-κB and GSK3-β signaling pathways to further enhance osteoclast differentiation [93]. In synovial tissue, LGR4 expression levels are significantly diminished in mice with traumatic osteoarthritis, and overexpression of LGR4 ultimately inhibits the proliferation of synovial cells and reduces joint inflammation [94]. Additionally, both Rspo1 and LGR4 levels are notably elevated in radiation-induced bone defects, and the RSPO1–LGR4 signaling pathway is activated to initiate self-repair mechanisms in BMSCs, thereby maintaining bone homeostasis [95]. Jang et al. found that a mutated RANKL protein acted as a competitive inhibitor of RANKL, binding exclusively to the receptor LGR4, which induced GSK-3β phosphorylation and suppressed the RANKL-RANK-TRAF6-NFATc1 signaling cascade, ultimately preventing osteoclast differentiation [96]. Furthermore, recent findings indicated that a novel RANKL variant induces the expression of LGR4 via the GSK3-β signaling pathway, thereby suppressing NFATc1 activity and inhibiting osteoporosis [97]. Taking these findings together, LGR4 plays a multifaceted role in a complex regulatory network of interactions among RSPO, RANKL, and Wnt/β-catenin signaling in bone homeostasis. However, the molecules that interact with LGR4 in regulating bone metabolism, including additional agonists/antagonists, other intracellular downstream signaling cascades, and neutralizing/activating antibodies, remain unclear.

## 5. Roles of Various Factors in the Development and Activation of Immune Cells

The immune system comprises a complex network of molecules, cells, and tissues that collaboratively protect the body from external and internal threats. RANK is recognized for its role in the development and growth of immune organs, such as the lymph nodes, thymus, bone marrow microenvironment, and intestinal tract [98,99]. In this context, LGR4, another RANKL receptor, has been identified as a modulator of immune responses and immune organ development, similar to the functions of other pleiotropic cytokines [100]. T cells produce RANKL, which subsequently leads to proliferation and activation via interleukin-4 (IL-4) and transforming growth factor (TGF) [101]. RANKL-mutant mice show normal spleen architecture and dendritic cell (DC) development; however, they display abnormalities in T cell differentiation and development [102,103]. In IL-2-knockdown mice, increased RANKL expression levels in T cells are associated with enhanced survival of intestinal CD11c+ DCs, resulting in bone loss and colitis [104]. In mice with CD40 downregulation, treatment with RANK-Fc inhibits T-cell-related immune responses and suppresses the survival of viruses and parasites, providing evidence that CD40L signaling may compensate for RANKL signaling [105]. Specific inhibition of RANKL effectively mitigates bone resorption and enhances immune responses by reducing regulatory T (Treg) cell activity, indicating an immunoregulatory role for this classical osteoclast mediator [106]. Blockade of RANKL activity through RANK-Fc protein or RANKL monoclonal antibody therapy results in a decrease in the number of Treg cells, which subsequently exacerbates type 1 diabetes [107]. Following the discontinuation of these anti-RANKL therapies, Treg cell numbers exhibit a slight increase, suggesting that alterations in immunomodulation are reversible and may persist longer than the rapidly reversible effects observed in the bone, commonly referred to as rebound bone resorption [108]. Consequently, the effect of RANKL on Treg cells may represent a feedback mechanism for immunomodulation. RANKL-LGR4 signaling is crucial for the activation and survival of DCs and T cells. LGR4 signaling in DCs downregulates the NF-κB and MAPK pathways, leading to the transcriptional activation of genes essential for survival and differentiation [109]. DCs are vital for antigen presentation and T cell activation [110]. RANKL treatment enhances the capacity of DCs to activate T cells, thereby promoting immune responses [111]. Additionally, RANKL signaling modulates T cell function, facilitating their activation and survival [98]. Both RANK and CD40, members of the TNFR family, activate similar intracellular signaling pathways in DCs and share binding sites for TRAF-family proteins [112]. However, CD40 signaling upregulates the expression of co-stimulatory and major histocompatibility complex (MHC) molecules in DCs [113].

RANKL has also been identified as a significant factor in the recruitment and organization of B cells within lymphoid tissues [114]. Follicular dendritic cells (FDCs) and marginal reticular cells produce the chemokine CXCL13, which is crucial for B cell recruitment [115]. RANKL signaling is integral to the development of FDCs and the subsequent production of CXCL13, as evidenced by the absence of B cells and FDCs in RANK-signaling-deficient mice [116]. This finding implies that RANKL is involved in both B cell recruitment and follicle organization in lymphoid tissues. B cell maturation and differentiation occur in proximity to osteoblasts, suggesting a close communication and interrelationship between these cell types [117]. Cytokines that influence bone metabolism, including TNF-α, IL-1β, and IL-13, as well as vascular cell adhesion molecules, directly impact B cell homing and differentiation [118,119]. Germline deletions of Tnfrsf11a (which encodes RANK) or Tnfsf11 (which encodes RANKL) compromise B cell development [120,121]. Additionally, mutations in TNFSF11A lead to a decrease in human immunoglobulin-secreting B cells [122]. The inhibition of systemic RANKL-LGR4 signaling can result in severe osteoporosis, which, in turn, leads to the loss of the bone marrow microenvironment necessary for B cell development. Impaired myeloid B cell development has also been observed in Rag1-deficient mice reconstituted with fetal liver cells from Tnfsf11-deficient mice, indicating the importance of LGR4 in the development of B cells from hematopoietic lineage cells [123]. However, conditional deletion of Tnfrsf11a specifically in B cells does not significantly affect B cell development or antibody production, suggesting that RANK is not essential for these processes in B cells [124].

LGR4 has been identified as a critical regulator of pancreatic β cell proliferation and survival in vivo under basal conditions and in response to stress [125]. GPCRs, including the GLP1 receptor (GLP1R), serve as essential mediators of various cellular processes and represent important therapeutic targets for a range of diseases, including diabetes [126]. Conditional β-cell-specific knockout of Glp1r results in the abrogation of insulin responses to both intravenous and intraperitoneal administration of GLP1 [127]. Barrella et al. demonstrated that the conditional β-cell-specific knockout of another GPCR member, β-arrestin-1, leads to compromised β-cell replication and function in insulin-resistant mice subjected to an obesogenic diet [128]. Thus, the pancreatic β-cell, a pivotal component in metabolic regulation, can be included among the cell types influenced by LGR4. This review highlights the necessity of preserving appropriate stoichiometry between the following two receptors: LGR4, which acts as a positive modulator, and RANK, which functions as a negative modulator of β-cell survival. Further investigations are needed to assess the therapeutic implications of this receptor and its potential ligands for diabetes.

## 6. Role of LGR4 in Osteoimmunology-Related Disease

Osteoimmunology is an interdisciplinary field that investigates the interactions between the skeletal and immune systems, which are crucial for understanding various bone-related pathologies, including osteoporosis and RA. This field emerged after the identification of LGR4 as a significant mediator of the communication between immune cells and osteoclasts, primarily regulating their differentiation and formation. Furthermore, the immune system modulates osteoclast activity through the secretion of cytokines, such as TNF-α, IL-1β, and IL-13. In addition to its role in bone regulation, LGR4 exerts immunoactive effects by promoting the production of Treg cells and influencing the activation and survival of DCs. Notably, co-stimulatory molecules initially characterized within the immune system, such as FcRγ and DAP12, are critical for RANK expression and osteoclast formation, underscoring the intricate interplay between these systems in the context of osteoimmunology [107]. Numerous diseases, including RA, osteoarthritis, periodontitis, osteoporosis, multiple myeloma, and metastatic bone tumors, exemplify the pathological relationship between immune and bone cells [129]. The most extensively studied condition is RA, which is a chronic inflammatory disease that can induce cartilage and bone destruction [130]. Although the precise pathogenesis of RA remains unclear, it is believed to be mediated by immune mechanisms [131]. Animal models of RA that lack RANKL expression exhibit joint inflammation without localized bone loss [132]. The secretion of IL-17 by Th17 cells stimulates synovial fibroblasts within the inflammatory milieu, leading to RANKL expression and subsequent osteoclastogenesis, which contributes to bone destruction [133]. Notably, Th17 cells that have lost Foxp3 expression and are deficient in IL-17 can also induce osteoclastogenesis [134]. LGR4 exhibits a pro-inflammatory role in models of RA [26]. The concurrent upregulation of LGR4 and TNF in double-transgenic mice accelerated the onset of the disease, resulting in severe arthritis characterized by significant increases in clinical and histological scores, aggressive pannus formation, extensive bone resorption, and substantial accumulation of inflammatory cells, predominantly of myeloid origin [135]. Additionally, TNF overexpression induces osteoclastogenesis independent of LGR4 at sites of inflammatory infiltration, whereas the combined effects of TNF and LGR4 exert systemic effects, affecting distal femoral regions [136]. Consequently, clinical data indicate that denosumab has a limited impact on joint inflammation but is beneficial for preventing bone destruction [137]. Therefore, it is advisable to consider its use in conjunction with other therapeutic agents, such as methotrexate or biologics, for the comprehensive management of immune-mediated diseases such as RA.

## 7. Conclusions and Prospects

Knowledge of the diverse physiological and pathological functions of LGR4 in various cellular processes provides a systematic and comprehensive understanding of its functional properties, thereby facilitating the development of new diagnostic biomarkers and therapeutic targets for various diseases. Interactions between LGR4 and its ligands, such as RSPO and RANKL, can activate downstream signaling pathways, including Wnt and other G-protein-coupled pathways. This indicates that LGR4 is involved in various mechanisms that modulate cellular responses in the bone in an immune-system-specific manner. The expression levels of LGR4 must be tightly regulated, as both insufficient and excessive levels can lead to detrimental conditions, such as osteoporosis, delayed puberty, obesity, and cancer. Furthermore, the endogenous ligands that activate LGR4 in association with the LGR4/cAMP/PKA signaling pathway are yet to be identified. Notably, the function of LGR4 through the recently discovered ligand RANKL has primarily been associated with the maintenance of bone tissue homeostasis; however, its role in immunoregulation remains largely unexplored. Therefore, future investigations should involve the experimental targeting of LGR4 in various bone- and immune-related diseases. More detailed mechanistic investigations are essential to elucidate the spatiotemporal profile of LGR4, which may pave the way for the development of novel molecular targeted therapies for such diseases. Such studies may uncover new signaling functions and physiological roles of LGR4, thereby providing additional avenues for therapeutic exploration. Additionally, research assessing the presence of LGR4-ECD in the circulation and its implications in both physiological and pathophysiological contexts may also hold therapeutic significance.

## Data Availability

All requests for raw and analyzed data and materials will be promptly reviewed to verify whether the request is subject to any intellectual property or confidentiality obligations by the corresponding author and Chosun University, Republic of Korea.

## Abbreviations

The following abbreviations are used in this manuscript:

BMSCs	Bone marrow stromal cells
BMD	Bone mineral density
BMP	Bone morphogenetic protein
CREB	cAMP response element-binding protein
cAMP	Cyclic adenosine monophosphate
DC	Dendritic cell
FDCs	Follicular dendritic cells
FZD	Frizzled
GPCR	G-protein-coupled receptor
GLP1R	GLP1 receptor
HSC	Hematopoietic stem cell
IL-4	Interleukin-4
LGR4	Leucine-rich repeat-containing G-protein-coupled receptor 4
LRR	Leucine-rich repeat
MHC	Major histocompatibility complex
OPG	Osteoprotegerin
PKA	Protein kinase A
PDK	Pyruvate dehydrogenase kinase
RANKL	Receptor activator of nuclear factor kappa-Β ligand
Treg	Regulatory T cell
SNP	Single nucleotide polymorphism
TLR	Toll-like receptor
TGF	Transforming growth factor
